# Effectiveness and Safety of a Probiotic-Mixture for the Treatment of Infantile Colic: A Double-Blind, Randomized, Placebo-Controlled Clinical Trial with Fecal Real-Time PCR and NMR-Based Metabolomics Analysis

**DOI:** 10.3390/nu10020195

**Published:** 2018-02-10

**Authors:** Maria Elisabetta Baldassarre, Antonio Di Mauro, Silvio Tafuri, Valentina Rizzo, Maria Serena Gallone, Paola Mastromarino, Daniela Capobianco, Luca Laghi, Chenglin Zhu, Manuela Capozza, Nicola Laforgia

**Affiliations:** 1 Neonatology and Neonatal Intensive Care Unit, Department of Biomedical Science and Human Oncology, Aldo Moro University of Bari, 70100 Bari, Italy; antonio.dimauro@uniba.it (A.D.M.); rizzo.vale@libero.it (V.R.); manuela.capozza@uniba.it (M.C.); nicola.laforgia@uniba.it (N.L.); 2 SIGENP (Italian Society of Pediatric Gastroenterology, Hepatology, and Nutrition), via Libero Temolo 4 (Torre UB), 20126 Milan, Italy; 3 Section of Hygiene, Department of Biomedical Science and Human Oncology, Aldo Moro University of Bari, 70100 Bari, Italy; silvio.tafuri@uniba.it (S.T.); serenagallone@gmail.com (M.S.G.); 4 Department of Public Health and Infectious Disease, “Sapienza” University of Rome, 00100 Rome, Italy; paola.mastromarino@uniroma1.it (P.M.); daniela.capobianco@uniroma1.it (D.C.); 5 Department of Agro-Food Science and Technology, University of Bologna, Piazza Goidanich 60, 47522 Cesena, Italy; l.laghi@unibo.it (L.L.); chenglin.zhu2@unibo.it (C.Z.)

**Keywords:** infantile colic, probiotics, metabolomics, microbiota

## Abstract

Introduction: To investigate the effectiveness and the safety of a probiotic-mixture (Vivomixx^®^, Visbiome^®^, DeSimone Formulation^®^; Danisco-DuPont, Madison, WI, USA) for the treatment of infantile colic in breastfed infants, compared with a placebo. Methods: A randomized, double-blind, placebo-controlled trial was conducted in exclusively breastfed infants with colic, randomly assigned to receive a probiotic-mixture or a placebo for 21 days. A structured diary of gastrointestinal events of the infants was given to the parents to complete. Samples of feces were also collected to evaluate microbial content and metabolome using fecal real-time polymerase chain reaction (qPCR) and Nuclear magnetic resonance (NMR)-based analysis. Study registered at ClinicalTrials.gov (NCT01869426). Results: Fifty-three exclusively-breastfed infants completed three weeks of treatment with a probiotic-mixture (*n* = 27) or a placebo (*n* = 26). Infants receiving the probiotic-mixture had less minutes of crying per day throughout the study by the end of treatment period (68.4 min/day vs. 98.7 min/day; *p* = 0.001). A higher rate of infants from the probiotic-mixture group responded to treatment (defined by reduction of crying times of ≥50% from baseline), on day 14, 12 vs. 5 (*p* = 0.04) and on day 21, 26 vs. 17 (*p* = 0.001). A higher quality of life, assessed by a 10-cm visual analogue scale, was reported by parents of the probiotic-mixture group on day 14, 7.1 ± 1.2 vs. 7.7 ± 0.9 (*p* = 0.02); and on day 21, 6.7 ± 1.6 vs. 5.9 ± 1.0 (*p* = 0.001). No differences between groups were found regarding anthropometric data, bowel movements, stool consistency or microbiota composition. Probiotics were found to affect the fecal molecular profile. No adverse events were reported. Conclusions: Administration of a probiotic-mixture appears safe and reduces inconsolable crying in exclusively breastfed infants.

## 1. Introduction

Infantile colic is a benign, self-limited process characterized by paroxysms of inconsolable crying. It affects approximately 10% to 40% of infants worldwide [1] associated with significant parental frustration, as well as repeated physician visits [2].

Infantile colic has been classified as a functional gastrointestinal disorder whose pathogenesis remains unknown, despite decades of research [3]. Consequently, various treatments have been tried to alleviate this condition [4].

Recent studies and preclinical data have suggested that changes of newborn intestinal microbiota can affect brain signaling systems related to pain, playing a pathogenetic role of infantile colic [5]. For this reason, dietary supplementation with probiotics has been suggested as a possible preventive [6] or therapeutic measure [7], by affecting perception centrally. Effects and safety of probiotics are highly strain-specific [8,9].

Despite data not being conclusive, at present, only *Lactobacillus reuteri DSM 17938* (*L. reuteri*) seems to increase colic treatment success in meta-analysis [10,11] and systematic reviews [12,13]. *L. reuteri* did not cause an increase in d-lactic acid beyond two weeks but d-lactic acidosis in infants who were fed lactic acid bacteria-containing products is still a concern in prolonged supplementation [13].

Other probiotics with a good safety profile have been evaluated for functional gastrointestinal disorders in pediatric patients. The high-concentrated multi-strain probiotic mixture, as specified below, has been proposed for the management of symptoms in children with irritable bowel syndrome (IBS) [14].

Our research group has recently suggested a possible preventive role in infantile colic by using this multi-strain probiotic mixture through maternal supplementation during late pregnancy and lactation [15]. This preliminary data in early infancy and pregnancy give good support for the use of this probiotic-mixture, in drop formulation, for treatment of infantile colic.

## 2. Materials and Methods

### 2.1. Study Design and Patients

This was a double-blind, randomized, placebo-controlled clinical trial (study registered at ClinicalTrials.gov: NCT01869426), performed to assess the efficacy and safety of a lyophilized high-concentration multi-strain probiotic mixture in reducing colic symptoms in exclusively breastfed infants.

The study was carried out between April 2015 and April 2016 in the Pediatric Outpatient Service of the Neonatology and Neonatal Intensive Care Unit (NICU) section of the Department of Biomedical Science and Human Oncology, of “Aldo Moro” University of Bari, Italy.

To be eligible for the study protocol, infants had to be healthy and well-fed, born at term, aged between 30 and 90 days, exclusively breastfed before and throughout all the study period, with a diagnosis of infantile colic (defined as crying episodes lasting three or more hours per day and occurring at least three days per week [16], within seven days prior to enrolment).

Exclusion criteria were: (a) major acute or chronic diseases; (b) gastrointestinal diseases and gastro-esophageal reflux; (c) use of probiotics/antibiotics the week before or during randomization; (d) gastrointestinal malformations; and (e) concurrent enrolment in other clinical trials.

Written informed consent was obtained from a parent/guardian, in accordance with the local ethics committee, which reviewed and approved the study protocol.

All infants enroled were randomized to receive, either oral probiotics or the placebo. Randomization was performed using a computer-generated two-treatment allocation sequence (nQuery Advisor v.7.0 software, Statistical Solutions Ltd., Cork, Ireland). To avoid disproportionate numbers of patients in each group, a randomization scheme was performed in blocks of four participants. Randomization codes were secured until all data were analyzed.

All participants, as well as scientific and medical personnel dedicated to the study, were blind to the group assignment. An independent person prepared the randomization schedule and took care of the packaging and labelling of the products.

Parents were requested to administer to their infants 10 drops of the probiotics, once a day, for 21 days directly in the mouth, preferably in the morning before feeding. Instructions for keeping and maintaining the product were also provided according to manufacturer indications. Parental compliance was assessed by a diary. The parents were also asked to return used bottles.

Even though maternal diet can influence colic frequency, no specific dietary restrictions during lactation were recommended, except to avoid any commercial products containing probiotics [17]. Parents were also instructed to avoid any other methods of managing infant colic.

The active study product was a medium chain triglycerides oil suspension containing a lyophilized high-concentration multi-strain probiotic mixture of four different strains of lactobacilli (*L. paracasei* DSM 24733, *L. plantarum* DSM 24730, *L. acidophilus* DSM 24735, and *L. delbrueckii subsp. bulgaricus* DSM 24734), three strains of bifidobacteria (*B. longum* DSM 24736, *B. breve* DSM 24732, and *B. infantis* DSM 24737), and one strain of *Streptococcus thermophilus* DSM 24731, produced at Danisco-Dupont, WI, USA and currently sold in Continental Europe, USA and Korea under the brand Vivomixx^®^, Visbiome^®^ and DeSimone Formulation respectively.

Ten drops of the formulation delivered a dose of 5 billion colony-forming units (CFU) of *L. paracasei* DSM 24733, *L. plantarum* DSM 24730, *L. acidophilus* DSM 24735, *L. delbrueckii* subsp. *bulgaricus* DSM 24734, *B. longum* DSM 24736, *B. breve* DSM 24732, *B. infantis* DSM 24737, and finally of *Streptococcus thermophilus* DSM 24731. Maltodextrine was used as an excipient. The medium chain triglycerides oil suspension complied with baby food requirements as per regulation 1881/2006 and directives 2006/141 and 2006/125.

The placebo was characterized by an identical formulation, but without any probiotic. The active and placebo products were identical and supplied in the same bottle, in order to maintain a double-blind status. All study products were kept refrigerated until use. The randomization codes were secured during each phase of the trial and opened only after final data analysis.

Both the active and placebo study products were donated by Prof De Simone/CD Investments who had no role in any phase of the study.

According to the study protocol, when an infant was referred with a diagnosis of infantile colic and fulfilled all criteria for enrolment, parents were asked to record colic symptoms for one week. If symptoms fulfilled the “infant colic” definition during that period, infants were enrolled in the study protocol.

### 2.2. Analysis of Infant Colic Symptoms

A structured diary to record gastrointestinal events, such as a feeding schedule, daily episodes of fussing/crying, the number of minutes of inconsolable crying per day, the number of bowel movements per day, and stool consistency (according to the Bristol Stool Form Scale for children) [18] was given to parents. The diaries used in this study were designed to be easy to use and to be suitable for a diverse sample of the population. “Fussing” was referred to elements of movement, facial expression, and voice that are usually interpreted as expressing a negative emotion.

Parents also recorded the times of administration of the study products, family quality of life, both at enrolment and at the end of the study, as well as any adverse events, such as constipation, vomiting, and skin reactions.

Quality of life was assessed by a 10-cm visual analogue scale with a numerical rating scale from 0 (worst possible well-being) to 10 (perfect well-being), as already described previously [19]. Parents were encouraged to contact the study personnel whenever needed.

On enrolment (day 0), a pediatric medical examination was performed and the following information was collected: (1) gestational age; (2) type of delivery; (3) birth weight; (4) anthropometric data at entry; (5) family history of gastrointestinal disease; and (6) family history of atopy.

Follow-up visits were scheduled at 7, 14, and 21 days after the beginning of the administration of study products, performed by the same referring pediatrician.

At the end of the study all the diaries were collected. Diary analysis and data entry were performed independently by two experienced investigators, both blinded from treatment allocation.

### 2.3. Analysis of Infant Stool Samples

To evaluate if probiotic supplementation showed a real impact on intestinal microbiota composition, two stool samples of infants were collected, once at enrolment and then 21 days after the beginning of the administration of study products. All samples were collected from diapers in sterile plastic tubes and stored at −80 °C until analysis. qPCR was used to quantify bifidobacteria and lactobacilli using genus-specific primers and conditions, as already described previously [20].

Each sample of feces was also prepared for proton nuclear magnetic resonance (^1^H-NMR) by mixing 80 mg of stool for 5 min with 1 mL of bi-distilled water and centrifuged at 4 °C and 18,630 *g* for 15 min. The supernatant (700 μL) was added to 100 μL of a D_2_O solution of 3-(trimethylsilyl)-propionic-2,2,3,3-d4 acid sodium salt (TSP) 10 mM, used as a reference for NMR chemical-shift, buffered at pH 7.00 by means of 1M phosphate buffer.

Each ^1^H-NMR spectrum was recorded at 298 K with an AVANCE III spectrometer (Bruker, Milan, Italy) operating at a frequency of 600.13 MHz. According to Ventrella et al. [21], a CPMG-filter composed of 400 180° pulses of 24 μs each, separated by 400 μs intervals, was employed to suppress the signals from broad resonances. Presaturation was applied to suppress the HOD residual signal. To acquire each spectrum, 256 transients were summed up, measuring 32 K data points spanning 7184 Hz of spectral window, with a 2.28 s acquisition time.

To obtain quantitative values by NMR, 5 s of recycle were chosen, in agreement with the relaxation time of the protons under investigation [22]. ^1^H-NMR spectra were baseline-adjusted by means of peak detection according to the “rolling ball” principle implemented in the baseline R package [23,24].

Each spectrum was then linearly corrected, so to make the baseline points randomly spread around zero. Signals were not manually aligned, which was different from previous investigations [25].

Probabilistic quotient normalization [26] was applied to the entire array of spectra to remove the effects of differences in water and fibers content among samples.

The signals were assigned by comparing their chemical shift and multiplicity with the Human Metabolome Database [27] and Chenomx software library (Chenomx Inc., Edmonton, AB, Canada, ver 8.1). Molecules with unknown chemical structures were also detected and quantified. Following Ventrella et al. [21], these molecules will be referred to throughout the text with an x followed by the chemical shift in ppm of their signal used for quantification (i.e., x–4.20).

### 2.4. Statistical Analysis

The primary outcome measures was the rate of responders and non-responders in improvement of colic symptoms. Success rate was defined by a reduction of the daily average crying time ≥50%, expressed in minutes.

Secondary outcome measures were: (1) average crying time per day; (2) parental quality of life; (3) any other gastrointestinal events; (4) anthropometrical evaluations; (5) side effects; (6) amount of lactobacilli and bifidobacteria in the stool samples of infants; (7) metabolomics evaluation of feces by means of ^1^H-NMR.

A reduction of the daily average crying time ≥50% was used to calculate the sample size that was 26 patients per group. For α = 0.05, β = 0.10, and an estimated SD within groups of 55%, 26 patients were needed per group [19]. Considering a potential dropout rate of 20%, thirty-three subjects per group were enroled.

Data from the first visit forms, diaries, and the results of the analysis of stool samples were reported in a database created by Google Drive software (Google ILC, Mountain View, CA, USA).

Statistical analyses were performed using Stata12MP (StataCorp LLC, College Station, TX, USA) and a per-protocol approach.

Quantitative variables with normal distribution were compared using the Student’s *t*-test. The Mann-Whitney U Test was used for non-normally distributed variables. Proportions were compared using the χ^2^ test or Fisher Exact Test, as appropriate.

Linear regression and logistic regression were performed to determine the effects of probiotic supplementation, vaginal delivery, family history of atopy and family history of gastrointestinal disease (determinants) on the duration of crying at 21 days, the total average crying minutes and the treatment response at day 14 and at day 21 (outcomes). In the logistic regression, the adjusted odds ratio, with 95% CI, were calculated and a z-score test was performed. Coefficiency with 95% CI and *t* values were calculated for linear regression. For all tests, a *p* value of <0.05 was considered as significant.

In the fecal metabolomics evaluation, molecules whose concentration varied in relation to the investigated treatments were looked for by calculating the T_21_–T_0_ differences and then they were compared by means of Mann-Whitney U test.

To highlight the underlying trends characterizing the samples, a robust Principal Component Analysis (rPCA) was built on the molecules concentration, centered and scaled to unity variance, according to Hubert [28].

For each PCA model, we calculated the scoreplot and the projection of the samples in the PC space, tailored to highlight the underlying structure of the data. We calculated the correlation plot, relating the concentration of each variable to the components of the PCA model, therefore, tailoring the samples to highlight the most important molecules in determining the trends emphasized by the scoreplot. The same subjects were followed along the entire experiment, giving rise to a repeated measurement data structure. Molecule concentrations at timepoint T_0_ were subtracted from every timepoint, as suggested by Ndagijimana [29].

## 3. Results

**Figure 1:**
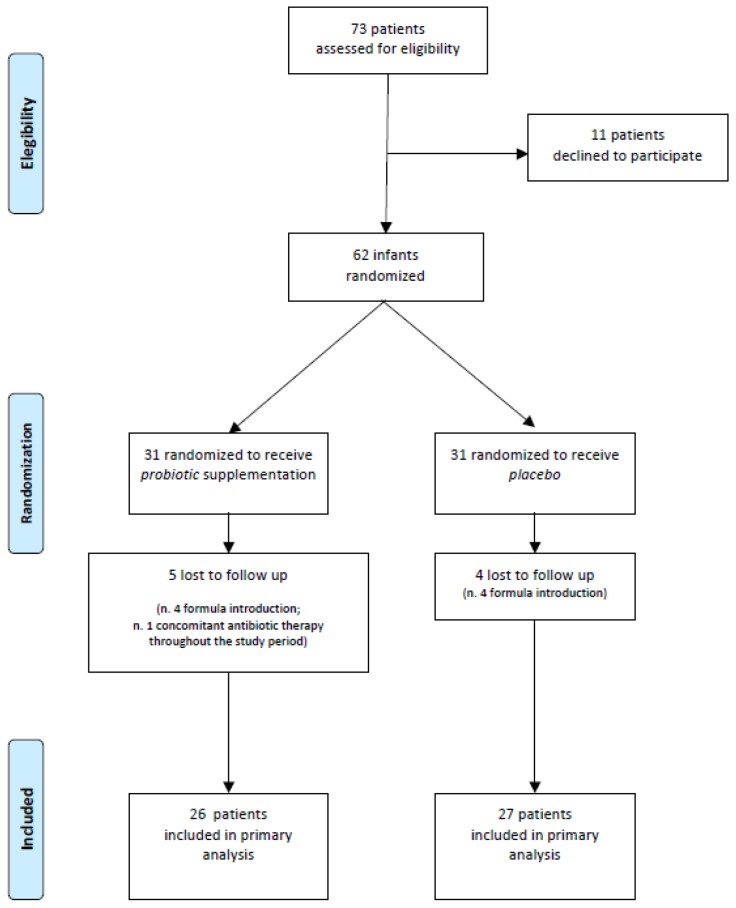
Study flow.

Of the 73 infants assessed for eligibility, seven refused to participate and 66 were enroled and randomized to receive placebo (*n* = 33) or probiotics (*n* = 33). After 21 days of intervention, 13 infants failed the follow-up, so that 53 (26 in the placebo group and 27 in the probiotics group) completed the study (Figure 1).

Nineteen samples (8 in the placebo group and 11 in the probiotics group) were evaluated with qPCR and metabolomics analysis. Characteristics of the infants at baseline are detailed in Table 1.

No significant differences were observed for age, sex, birth weight, and anthropometrical data. Vaginal birth, family history of gastrointestinal diseases, and atopy were significantly higher in probiotic group. At baseline, the average duration of crying was similar for the two groups.

A higher and statistically significant rate of treatment success was found in the probiotic group, compared to the placebo group on day 14 and on day 21 (Table 2).

The total average crying minutes throughout the 21 days of the study were significantly less in the probiotic group compared to the placebo group (Table 3).

Parents of the infants of the probiotic group reported an improved family quality of life compared to the placebo group on day 14 and on day 21. Bowel movements and stool consistency did not differ between the two groups, as well as there being no differences of growth. Both the probiotic and the placebo were well tolerated and no side effects were reported.

Multivariate analyses confirmed that the probiotic group showed a reduction of crying on day 21 (coeff. −41.7; *t* = −3.92; 95%CI: −63.2–−20.3; *p* = 0.00) and a reduction also of the total average crying minutes throughout the study period (coeff. −77.4; *t* = −2.55; 95% CI: −138.3–−16.4; *p* = 0.01). The treatment response on day 14 (aOR = 4.87; 95% CI = 1.1–21.3; *z* = 2.10; *p* = 0.03) and on day 21 (aOR = 28.0; 95% CI = 1.76–444.3; *z* = 2.36; *p* = 0.01) were associated with probiotic administration, and with no confounding effects from vaginal delivery, family history of gastrointestinal disease, and a family history of atopy (*p* > 0.05).

**Figure 2:**
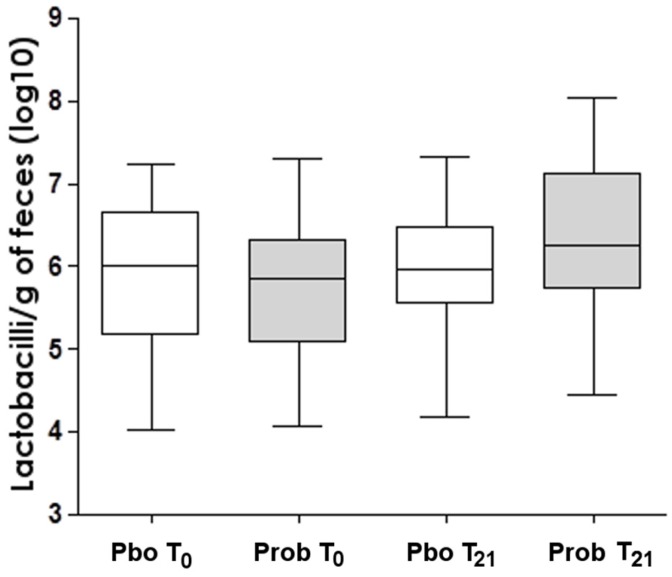
Lactobacilli concentration in feces of placebo (pbo)- and probiotics (prob)-supplemented infants.

Total bacteria, lactobacilli and bifidobacteria, in the sample feces were not different between infants treated with the probiotics and the placebo. However, a slight increase (*p* = 0.053) of lactobacilli in stool samples of the probiotic supplemented infants was observed (Figure 2).

To understand how probiotic supplementation could affect the fecal molecular profile, a metabolomics investigation on feces by means of ^1^H-NMR was established. Fifty-nine molecules could be quantified pertaining to the chemical groups of amino acids, short chain fatty acids, organic acids and monomeric carbohydrates. Another 27 molecules could be quantified, with a partially unclear structure and, therefore, denoted with the location in the NMR spectrum, expressed in ppm. To highlight the differences in the effects of probiotic treatment with respect to the placebo, we calculated the T_21_–T_0_ difference for each molecule on an individual basis and we compared the differences by a two-tailed Mann-Whitney U test. The evolution of the fecal metabolome differed for 12 molecules, as detailed in Table 4.

Interestingly, 10 out of 12 molecules showed opposite trends for the two groups, with acetate and methylamine representing the only exceptions. Consequently, samples at T_21_ constituted of two distinct groups according to the treatment, as it could be inferred also form the statistical significance (*p* < 0.01) of the intragroup/intergroup samples distance in the 12 dimensions space.

**Figure 3:**
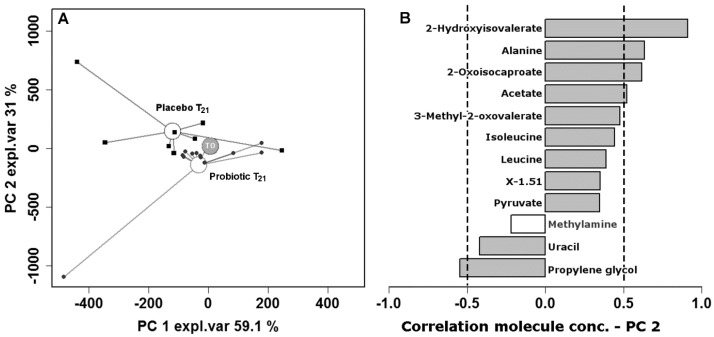
rPCA model built on the space, constituted by the concentration of the molecules listed in Table 4. In the scoreplot (**A**), samples from people treated with the placebo and the probiotics are represented with squares and circles respectively. The wide, empty circles represent the median of the samples at the various time-points. Samples at T0 from the two groups are superimposed. In the barplot (**B**), describing the correlation between the concentration of each molecule and its importance over PC 2, gray bars highlight statistically significant correlations (*p* < 0.05).

To have an overview of such findings, we calculated an rPCA model on the centered and scaled concentrations of these molecules (Figure 3).

To consider the paired structure of the experiment, we subtracted the concentration of the molecules at timepoint T_0_ from every timepoint. This is why, in the scoreplot (Figure 3A), the two groups at T_0_ appear superimposed and with scores along PC 1 and PC 2 very close to 0.

The median scores of both groups at T_21_ appear at negative values along PC 1. Such PC, therefore, allows us to focus on the changes occurring to the metabolome of the infants upon growing.

Along PC 2, contrastingly, samples from the placebo group appear at positive scores, while samples from the probiotic group appear separated from the previous (*p* < 0.01), with negative scores. PC 2, therefore, gives a holistic view of the different responses of infants, to the two treatments, evidenced by Table 4 on a molecule-by-molecule basis. From this perspective, it is of importance to notice that along PC 2, samples at T_21_ appear differently from samples at T_0_ only for infants treated with probiotics (*p* < 0.01). The molecules that mostly contributed to the trends (Figure 3B) were 2-hydroxyisovalerate, alanine and 2-oxoisocaproate, increasing only in subjects treated with the placebo. Acetate increased mainly in subjects treated with the placebo and propylene glycol, as well as increasing in subjects treated with probiotics.

## 4. Discussion

Our double-blind, placebo-controlled randomized trial suggests that the use of a mixture of eight probiotic strains, in exclusively-breastfed infants with infantile colic, reduces crying.

Recent studies have evaluated the role of microbiota in the development of gastrointestinal functions [30]. Microbiota interact with the gut-brain axis and can modify gut sensory and motor functions, emitting and receiving many signals to and from the brain. We can speculate that a modified microbiota colonization of the gastrointestinal tract could result in changes of this bidirectional interrelation with a possible pathogenetic role in infantile colic [31].

According to this, microbiota modulation through probiotic administration may be beneficial. Roos et al. [32] evaluated, in a randomized controlled trial, fecal samples of infants affected by colic, after *L. reuteri* DSM17938 or placebo administration and found that a decrease of symptoms in responders was related to the increase of *Bacteroidetes*. For this strain, a recent metanalisis [11], showed for infants with colic supplemented with probiotic, a reduction in crying and/or fussing duration of 46 min per day at day 21. In our multivariate analyses, the probiotic group showed a reduction of crying on day 21 of 41 min per day. To the best of our knowledge, this is the first trial aiming to assess safety and efficacy of a mixture of probiotics for the treatment of infantile colic in exclusively breastfed infants. Kianifar et al. showed that a mixture of seven probiotic strains plus prebiotic significantly improved colic symptoms in comparison with the placebo in breastfed infants [33]. Our findings are consistent with this study, although Kianifar et al. used a probiotic mixture plus prebiotic (FOS).

The effects of probiotics are strictly related to type and number of strains, dosage and duration of intervention, study population and environmental background, so any therapeutic effects have to be considered specific. Moreover, regarding any probiotic mixture, there are concerns about the possibility of inhibitory effects between different probiotic strains that may reduce efficacy, as demonstrated in vitro [34].

Despite infantile colic being considered a self-limited disease, different studies have shown that infants with colic have an increased risk of developing recurrent abdominal pain [35,36].

Our data supported the hypothesis that the impact of probiotics on gut microbiota may not involve only changes in intestinal bacteria composition but play a pivotal role in intestinal bacterial metabolism. In fact, despite our probiotic supplementation not modifing the amount of lactobacilli and bifidobacteria in the infants’ microbiota, the observation from a metabolomics perspective showed that the fecal molecular profile differed in connection with the treatments. In particular, this applied to the placebo group where alanine and two organic acids, 2-hydroxyisovalerate and 2-oxoisocaproate, both involved in valine, leucine, and isoleucine metabolism, were highly increased.

Elevated levels of alanine were found also in adults with IBS [37] and the two organic acids are thought to have a role in the signaling pathways of the neuro-immune system of the gut [38].

Furthermore, the placebo group expressed significantly higher levels of acetate, one of the main short chain fatty acids (SCFAs) produced during microbiota fermentation [39], whose fecal level is associated with worse gastrointestinal symptoms in adult patients with IBS [40].

In the present investigation, propylene glycol appeared as the clearest biomarker of subjects supplemented with probiotics. Propylene glycol is normally found in newborn feces and, interestingly, a higher concentration has been found in the feces of breast-fed infants in comparison with formula-fed ones, which suggesting a beneficial effect for this molecule [41].

According to Gonzalez et al. [42], this is due to an upregulation of lactaldehyde reductase, linked to the peculiar oligosaccharides composition on human milk, that uses L-fucose and L-rhamnose as substrates. Fucosylated milk oligosaccharides are thought to diminish colon motor contractions [43]. To the best of our knowledge, there are no reports on the effects of propylene glycol on the gastrointestinal system of humans. However, studies on rats suggested that propylene glycol may influence intestinal digestive and absorptive functions [43].

We are awake of some limitations. First, we used a nonvalidated diary of infant crying duration. Thus, we fully relied on parents’ reports on the duration of crying recorded, and mothers filled in every hour throughout the day to minimize recall bias. Second, we are not able to report on potential counfounders, such as maternal depression, maternal diets and early use of probiotic/antibiotic.

In conclusion, we speculate that the probiotic biological effects in this study are linked to microbial metabolomics with respect to the ability to colonize the gastrointestinal tract. Despite further studies being needed to fully understand these aspects, fucosylated milk oligosaccharides and their microbial degradation products seem to play a key role.

## 5. Conclusions

Daily supplementation with a specific, high-concentration probiotic preparation to breastfed infants modulates infantile colic symptoms by the end of treatment (21 days). Although colic is generally a self-limiting condition, a variety of management modalities have been proposed to decrease parental anxiety, frustration, and stress, as well as medical interventions and increased risk for developing recurrent abdominal pain. Further studies are needed to confirm that this specific probiotic mixture may be used for infantile colic treatment.

## Figures and Tables

**Table 1:** Demographical characteristics at enrolment.

	Placebo (*n* = 26)	Probiotic (*n* = 27)	*p* Value
Gestational age (week)	38.5 ± 1.8	39.0 ± 1.6	0.25 *
Male	13 (50.0)	18 (66.7)	0.21 °
Vaginal	7 (26.9)	15 (55.6)	0.03 °
Birth weight (g)	3156.5 ± 492.1	3367.9 ± 478.5	0.12 *
Age at enrolment (day)	40.9 ± 21.7	37.7 ± 17.2	0.56 *
Weight at enrolment (g)	4556.9 ± 985.6	4442.0 ± 691.9	0.62 *
Length at enrolment (cm)	55.1 ± 4.1	54.8 ± 2.6	0.75 *
Head circumference at enrolment (cm)	37.8 ± 2.2	37.9 ± 1.8	0.75 *
Family history of gastrointestinal disease	2 (7.7)	9 (33.3)	0.02 °
Family history of atopy	7 (26.9)	15 (55.6)	0.03 °
Duration of crying, min/day at baseline	211.6 ± 31.6	226.2 ± 31.0	0.09 ^†^

* Student *t*-test; ° χ^2^ test; ^†^ Mann-Whitney test; Results are expressed as mean ± SD (standard deviation) or *n* (%).

**Table 2:** Primary outcome.

	Placebo (*n* = 26)	Probiotic (*n* = 27)	*p* Value
Responder vs. non responder, *n* (%)			
Day 7	0 (0.0)	3 (11.1)	0.24 ^∆^
Day 14	5 (19.2)	12 (44.4)	0.04 °
Day 21	17 (65.4)	26 (96.3)	0.00 °

° χ^2^ test; ^∆^ Fisher exact test.

**Table 3:** Secondary outcomes.

	Placebo (*n* = 26)	Probiotic (*n* = 27)	*p* Value
Duration of crying, (mean ± SD) min/day			
Baseline	211.6 ± 31.6	226.2 ± 31.0	0.09 ^†^
Day 7	163.7 ± 35.9	152.2 ± 32.0	0.23 ^†^
Day 14	128.3 ± 36.6	112.3 ± 29.7	0.28 ^†^
Day 21	98.7 ± 39.2	68.4 ± 28.2	0.00 ^†^
Total crying/21 days, min	391 ± 96.9	333 ± 71.2	0.00 *
Family quality of life (mean ± SD)			
Baseline	5.8 ± 2.3	5.5 ± 2.1	0.28 *
Day 7	7.1 ± 1.5	6.5 ± 1.3	0.11 *
Day 14	7.1 ± 1.2	7.7 ± 0.9	0.02 *
Day 21	6.7 ± 1.6	8.3 ± 1.0	0.00 *
Bristol Scale Score (*n*, % score = 7)			
Baseline	12 (46.2)	8 (29.6)	0.32 °
Day 7	14 (53.9)	9 (33.3)	0.13 °
Day 14	12 (46.2)	11 (40.7)	0.36 °
Day 21	10 (38.5)	11 (40.7)	0.86 °
Mean Bowel movement per day (mean ± SD)			
Baseline	4.3 ± 2.4	3.4 ± 2.6	0.41 *
Day 7	3.8 ± 2.5	2.9 ± 2.1	0.35 *
Day 14	3.2 ± 2.4	2.6 ± 1.8	0.48 *
Day 21	3.3 ± 2.4	2.6 ± 1.9	0.27 *
Weight gain g/day (mean ± SD)			
Day 7	38.2 ± 27.0	55.8 ± 43.7	0.20 ^†^
Day 14	32.2 ± 10.5	21.3 ± 32.1	0.10 ^†^
Day 21	33.7 ± 26.2	31.7 ± 49.5	0.96 ^†^
Length gain cm/w (mean ± SD)			
Day 7	0.8 ± 1.1	1.2 ± 2.9	0.58 ^†^
Day 14	1.1 ± 1.3	1.7 ± 2.8	0.83 ^†^
Day 21	2.2 ± 1.1	2.6 ± 2.6	0.61 ^†^
Cranial Circumference gain cm/w (mean ± SD)			
Day 7	0.3 ± 0.6	0.5 ± 0.8	0.76 ^†^
Day 14	0.8 ± 0.8	0.9 ± 0.9	0.69 ^†^
Day 21	1.3 ± 0.6	1.1 ± 0.8	0.47 ^†^

* Student *t*-test; ^†^ Mann-Whitney test; ° χ^2^ test.

**Table 4:** Concentration (mM) of the molecules whose T_21_–T_0_ value was statistically different (*p* < 0.1) between subjects treated with placebo and probiotic.

	T_0_		T_21_–T_0_		
	Placebo	Probiotic	Placebo	Probiotic	*p* Value
Organic acids					
2-Oxoiso-caproate	4.29 × 10^−6^ ± 5.42 × 10^−6^	1.94 × 10^−5^ ± 1.94 × 10^−5^	1.80 × 10^−5^ ± 1.68 × 10^−5^	−4.11 × 10^−6^ ± 1.90 × 10^−5^	2.54 × 10^−3^
Pyruvate	2.86 × 10^−4^ ± 3.50 × 10^−4^	5.32 × 10^−4^ ± 5.32 × 10^−4^	8.57 × 10^−4^ ± 7.08 × 10^−4^	−2.60 × 10^−6^ ± 6.41 × 10^−4^	1.57 × 10^−2^
Acetate	2.80 × 10^−2^ ± 2.00 × 10^−2^	2.83 × 10^−2^ ± 2.83 × 10^−2^	2.33 × 10^−2^ ± 2.16 × 10^−2^	2.31 × 10^−3^ ± 2.91 × 10^−2^	9.08 × 10^−2^
2-Hydroxyiso-valerate	3.60 × 10^−5^ ± 3.67 × 10^−5^	2.18 × 10^−4^ ± 2.18 × 10^−4^	5.97 × 10^−6^ ± 4.16 × 10^−5^	−6.81 × 10^−5^ ± 1.57 × 10^−4^	9.08 × 10^−2^
Amino acids					
Alanine	2.56 × 10^−3^ ± 1.97 × 10^−3^	2.19 × 10^−3^ ± 2.19 × 10^−3^	1.52 × 10^−3^ ± 2.38 × 10^−3^	−2.07 × 10^−4^ ± 1.35 × 10^−3^	3.28 × 10^−2^
Leucine	8.53 × 10^−4^ ± 4.31 × 10^−4^	4.73 × 10^−4^ ± 4.73 × 10^−4^	3.91 × 10^−4^ ± 1.11 × 10^−3^	−2.54 × 10^−4^ ± 4.54 × 10^−4^	7.54 × 10^−2^
Isoleucine	3.87 × 10^−4^ ± 1.94 × 10^−4^	2.28 × 10^−4^ ± 2.28 × 10^−4^	1.08 × 10^−4^ ± 3.45 × 10^−4^	−1.55 × 10^−4^ ± 2.37 × 10^−4^	6.20 × 10^−2^
Alcohols					
Propylene glycol	9.79 × 10^−2^ ± 2.44 × 10^−1^	2.84 × 10^−3^ ± 2.84 × 10^−3^	−8.92 × 10^−2^ ± 2.42 × 10^−1^	2.54 × 10^−3^ ± 9.53 × 10^−3^	5.06 × 10^−2^
Others					
Uracil	2.15 × 10^−4^ ± 2.29 × 10^−4^	6.79 × 10^−5^ ± 6.79 × 10^−5^	−1.20 × 10^−4^ ± 2.23 × 10^−4^	1.93 × 10^−5^ ± 7.10 × 10^−5^	1.57 × 10^−2^
Methylamine	3.00 × 10^−5^ ± 5.46 × 10^−5^	7.52 × 10^−6^ ± 7.52 × 10^−6^	−2.19 × 10^−5^ ± 5.44 × 10^−5^	5.89 × 10^−6^ ± 1.26 × 10^−5^	6.20 × 10^−2^
3-Methyl-2-oxo-valerate	1.36 × 10^−5^ ± 1.25 × 10^−5^	2.53 × 10^−5^ ± 2.53 × 10^−5^	3.63 × 10^−5^ ± 4.16 × 10^−5^	7.76 × 10^−6^ ± 2.10 × 10^−5^	9.08 × 10^−2^
x-1.51	1.80 × 10^−4^ ± 3.98 × 10^−4^	1.81 × 10^−4^ ± 1.81 × 10^−4^	1.20 × 10^−4^ ± 4.57 × 10^−4^	−2.89 × 10^−5^ ± 1.25 × 10^−4^	1.57 × 10^−2^

## Abbreviations

IBS	irritable bowel syndrome
CFU	colony forming units
NICU	Neonatal Intensive Care Unit
^1^H-NMR	Proton Nuclear Magnetic Resonance
SCFAs	short chain fatty acids
